# P-675. Online Clinician Education Curriculum Results in Performance Changes in Clinical Practice for Recommending Available Respiratory Syncytial Virus Vaccines in Older Adults

**DOI:** 10.1093/ofid/ofae631.871

**Published:** 2025-01-29

**Authors:** Arun S Nair, Catherine Capparelli, Iwona Misiuta

**Affiliations:** Medscape Education, TARRYTOWN, New York; Medscape Education, TARRYTOWN, New York; Medscape Education, TARRYTOWN, New York

## Abstract

**Background:**

This study examined whether online continuing medical education (CME/CE) focused on evidence-based best practices for recommending available respiratory syncytial virus (RSV) vaccines would result in improved understanding of adults disproportionally affected by RSV, the clinical guidance of available vaccines, and communication strategies to encourage older adults to get vaccinated for RSV.

**Methods:**

Clinicians who participated across a curriculum of 3, live and online, 30-minute CME/CE video discussions between experts. Performance was assessed by inviting learners to complete a survey 30-60 days post-education, identifying practice changes and the degree to which clinicians experience barriers to those changes. The data was collected from 12/29/2023 to 04/04/2023.

**Results:**

There were 13,232 learners consisting of PCPs, Pharmacists, Advanced Practice Providers, and others who completed the survey. The analyses from the program showed that ∼90% of learners made a practice change or had practices reinforced due to education. Five key practice changes to recommending available RSV vaccines included:

Educate eligible patients/caregivers on the risk of RSV in older adults (93% PCPs, 93% Pharm, 95% NPs/Nurses, 92% PAs)

Recommending available RSV vaccines for older adults (93% PCPs, 93% Pharm, 95% NPs/Nurses, 93% PAs)

Use alternative communication methodologies with patients (15% PCPs, 21% Pharm, 14% NPs/Nurses, 16% PAs)

Collaborate with different members of the patient’s care team (11% PCPs, 22% Pharm, 13% NPs/Nurses, 16% PAs)

Some of the practice barriers identified by the learners include:

Lack of clear recommendations from evidence-based guidelines

Lack of time to educate patients on the importance of getting vaccinated for RSV

Patient concerns/questions regarding available RSV vaccines

Organizational limitations to providing the vaccine

**Conclusion:**

The practice changes identified in this assessment provide compelling evidence that participation in online CME/CE prompts adoption of changes in practice related to RSV vaccination. Future education is needed to address the barriers identified in this assessment.

**Acknowledgement**:

This education was supported by an independent educational grant from Pfizer Inc. and in partnership with Immunize.org

**Disclosures:**

**All Authors**: No reported disclosures

